# Long-Term Intraocular Pressure Fluctuation and Epiretinal Membrane in Patients with Glaucoma or Glaucoma Suspect

**DOI:** 10.3390/jcm13041138

**Published:** 2024-02-17

**Authors:** Kyoung In Jung, Jiyun Lee, Da Young Shin, Chan Kee Park

**Affiliations:** 1 College of Medicine, The Catholic University of Korea, Seoul 06591, Republic of Korea; ezilean@hanmail.net (K.I.J.); lemonssung@hanmail.net (J.L.); eye-sdy1107@hanmail.net (D.Y.S.); 2 Department of Ophthalmology, Seoul St. Mary’s Hospital, Seoul 06591, Republic of Korea; 3 Department of Ophthalmology, Eunpyeong St. Mary’s Hospital, Seoul 03312, Republic of Korea

**Keywords:** glaucoma, intraocular pressure fluctuation, epiretinal membrane

## Abstract

**Background**: A relationship between glaucoma and epiretinal membrane (ERM) has been suggested previously. We investigated the association between intraocular pressure (IOP) fluctuation and idiopathic ERM in patients with glaucoma or glaucoma suspect. **Methods**: Among patients with glaucoma or glaucoma suspect, data from 43 patients with ERM and 41 patients without ERM were reviewed and analyzed in this retrospective study. The long-term fluctuation of IOP was defined based on the standard deviation of IOP across all visits. **Results**: Patients with ERM were older and had a higher SD of IOP and a higher proportion of having a history of cataract surgery and greater macular thickness (*p* = 0.018, 0.049, 0.013, and <0.001, respectively). In multiple logistic regression analysis, the high-IOP-fluctuation group was associated with the presence of ERM (*p* = 0.047). Among patients with ERM, eyes with stage-3 or -4 ERM had worse visual field defects based on mean deviation than those with stage-1 or -2 ERM (*p* = 0.025). **Conclusions**: Long-term IOP fluctuation was associated with idiopathic ERM in patients with glaucoma or glaucoma suspect. Idiopathic ERM could serve as a biomarker for long-term IOP fluctuation in glaucoma patients, particularly in clinics where measuring long-term IOP fluctuation during the first visit is not feasible due to its time-consuming nature.

## 1. Introduction

Idiopathic epiretinal membrane (ERM) is a pathologic preretinal fibrocellular tissue [1]. Several studies have demonstrated that ERM is composed mainly of glial cells, fibroblasts, hyalocytes, and an extracellular matrix [1,2]. Old age, history of cataract surgery, and diabetes have been identified as risk factors for idiopathic ERM [3,4]. The exact pathogenesis of ERM has not been confirmed, even though it is considered to be preceded by posterior vitreous detachment (PVD) [1,5]. Several studies have reported an association between ERM and glaucoma [6,7,8]. Eyes with early glaucoma had a higher probability of having ERMs than those with glaucoma suspect or control subjects [7]. In patients with primary open-angle glaucoma (POAG) and a unilateral ERM, the eyes with ERM had more severe glaucomatous visual field (VF) damage than the eyes without ERM [6]. Mechanical stress on the inner retina induced by ERM traction might induce retinal nerve fiber layer (RNFL) damage [6]. Microcystoid macular changes are frequently found in eyes with both ERM and glaucoma [9]. Pars plana vitrectomy with epiretinal and internal limiting membrane peel was not effective in the resolution of microcystoid macular changes in patients with ERM and accompanying glaucoma [9]. Therefore, the characteristics associated with glaucoma itself might play a role in the pathogenesis of ERM in patients with glaucoma, distinct from subjects with only ERM and no evidence of glaucoma.

Elevated intraocular pressure (IOP) is the major risk factor for glaucomatous optic neuropathy [10]. IOP is not a fixed value and shows diurnal or long-term fluctuations [11]. Eyes with glaucoma showed greater IOP fluctuation than those without glaucoma in several studies [12,13,14,15]. IOP fluctuations have been found to be one of the factors associated with the progression of glaucoma, even though there have been controversies [16,17,18,19]. In an animal model undergoing intermittent IOP elevations, an upregulation of astrocyte reactivity was found in the optic nerve head [20]. There is a possibility that IOP fluctuations also have an association with gliosis in the retina. A reactive gliosis is a phenotype of ERM because the formation of ERM involves glial proliferation in response to retinal injury or disease involving inflammatory and glial cells [21,22].

Taking the previous findings into account, there is a likelihood that IOP fluctuation might be associated with ERM in patients with glaucoma. In this study, we investigated the relationship between long-term IOP fluctuation and early to-late-stage ERMs in patients with glaucoma or glaucoma suspect. In addition, intereye comparison was performed to determine the ocular factors associated with ERMs in patients with bilateral glaucoma and unilateral ERMs. Given that IOP is intricately linked to the dynamics of aqueous humor production and outflow, and the autonomic nervous system plays a role in regulating these dynamics, we hypothesize that IOP fluctuation may be influenced by an imbalance in the autonomic nervous system [23]. To further investigate this hypothesis, we performed a subanalysis of parameters related to heart rate variability and IOP fluctuation.

## 2. Materials and Methods

The Institutional Review Board of the Catholic University of Korea, Seoul, Korea, approved this retrospective observational study and waived the need for written informed consent because of its retrospective design. This study followed the tenets of the Declaration of Helsinki. Data from patients with a diagnosis of primary open-angle glaucoma or glaucoma suspect and idiopathic ERM at the glaucoma clinic of Seoul St. Mary’s Hospital between 2008 and 2019 were collected. Data from patients with glaucoma or glaucoma suspect and without ERM who visited the glaucoma clinic from March 2009 to May 2009 and were followed up over a period of at least 4 years were collected. VF mean deviation (MD)-matched patients without ERM were selected. Inclusion criteria were an open angle and axial length less than 28 mm. Patients with a history of or current uveitis, brain disease that could affect vision, or other vision-threatening retinal diseases except ERM, such as retinal vein obstruction, central serous chorioretinopathy, proliferative diabetic retinopathy, macular degeneration, or retinal detachment, were excluded.

Eyes with a glaucomatous optic disc such as rim loss, notching, and RNFL defects with corresponding VF damage were diagnosed as having glaucoma. Eyes with glaucomatous structural change in the absence of VF damage were diagnosed as glaucoma suspect.

All participants underwent a full ophthalmic examination, including Goldmann applanation tonometry, central corneal thickness and axial length measurements, gonioscopic assessments, red-free RNFL photography, and stereoscopic optic disc photography.

Long-term IOP fluctuation was determined as the standard deviation (SD) of all IOP values during treatment [24]. Low- and high-IOP-fluctuation groups were defined based on the median SD of IOPs. Peak and trough IOPs were defined as the maximum and minimum IOPs during treatment, respectively. The IOP range was calculated as the difference between the maximum and minimum IOPs. At the first visit to our clinic, all patients underwent blood pressure measurement in the sitting position using a standard automated blood pressure cuff after a 5 min rest.

### 2.1. Optical Coherence Tomography

With Cirrus SD-OCT version 6.0 (Carl Zeiss Meditec, Inc., Dublin, CA, USA), peripapillary RNFL thickness was measured using the Optic Disc Cube 200 × 200 scan mode, and the macular thickness was determined through macular thickness analysis using a macular cube 512 × 128 scan mode. The protocol by which peripapillary RNFL thickness was assessed was previously described in detail [25,26]. The presence of ERM was based on spectral domain-OCT as irregular, hyperreflective lines on the internal limiting membrane [27]. The ERM stage was determined as one of four stages according to the staging system suggested by Govetto et al. [28]. Among eyes with ERM, few eyes were classified as stage 1 or 4 ERM. The comparison according to the ERM stages was performed between eyes in the early ERM stage groups (stage 1 or 2 ERM) and eyes in the late ERM stage groups (stage 3 or 4 ERM). The macular thickness map displays macular thickness as the Early Treatment Diabetic Retinopathy Study (ETDRS) map. The macular thickness measured in the center circle with a 1 mm diameter was adopted in this study. Microcystic macular changes were indicated when following findings were detected: multiple, small hyprorefective roundish-elliptical cystoid spaces, without the presence of cyst wall, located in the inner nuclear layer and not confluent with cystoic spaces in other retinal layers [9]. Only images with signal length >6 and without misalignment or segmentation error were included.

### 2.2. Visual Field Testing

All patients underwent an SAP 24-2 test with a Humphrey field analyzer (Carl Zeiss Meditec, Dublin, CA, USA), using the Swedish interactive threshold algorithm standard strategy. Glaucomatous VF defects were defined as clusters of ≥3 points having sensitivities <5% of the normal population on the pattern deviation plot. One of the abnormal points should have a sensitivity <1% of the normal population. Mean deviation (MD) and pattern standard deviation were analyzed. Reliable VFs were considered those with <20% fixation losses, false positives, or false negatives.

### 2.3. Heart Rate Variability (HRV) Evaluation

In the glaucoma clinic, echocardiography was routinely performed on patients with normal tension glaucoma (NTG) if the patients agreed. An experienced technician performed the echocardiogram for 5 min, and the signals were transmitted to a Medicore Heart Rate Analyzer (SA-3000P, Medicore, Seoul, Republic of Korea). The standard deviation of the NN-interval (SDNN) index was obtained by measuring the normal R-R interval of the QRS complex in the echocardiogram.

The SDNN is affected by the autonomic nervous system. The low frequency (LF) component is considered to be a quantitative marker for sympathetic modulation, even though some studies regard LF as reflecting both sympathetic and parasympathetic activity [29]. The high frequency (HF) band, known as the respiratory band, represents parasympathetic activity [30].

### 2.4. Statistical Analysis

The program SPSS for Windows version 23.0 (SPSS Inc., Chicago, IL, USA) was adopted for all statistical analysis. For interindividual comparison, the Student’s *t*-test was used for continuous variables and the chi-square test for categorical variables. In the subgroup analysis on patients with available heart rate variability data, the Mann–Whitney U test was adapted for comparison between groups because the normality assumption was violated statistically, according to the Shapiro–Wilk test. For intereye comparison with a smaller number of subjects, the Wilcoxon signed rank test was performed because the normality assumption was not satisfied. Multiple logistic regression analysis was performed to determine the factors related to the presence of ERM in patients with glaucoma or glaucoma suspect. Factors with a difference of *p* value less than 0.05 between the two groups were included into the multiple logistic regression analysis. *p* < 0.05 was regarded to indicate statistical significance.

## 3. Results

Of a total of 84 patients with glaucoma (*n* = 77) or glaucoma suspect (*n* = 7), 41 patients had ERM, and 43 patients did not have ERM. Among glaucoma patients, 61 subjects (79.2%) were diagnosed as NTG and 16 patients (20.8%) as POAG. Seventy-two patients (85.7%) were using glaucoma eyedrops. Among the 41 patients with ERM, 9 patients were classified as stage 1 ERM, 18 patients as stage 2 ERM, 13 patients as stage 3 ERM, and only 1 patient as stage 4 ERM. Of the 41 patients with ERM, only one patient underwent surgical treatment for ERM. The median value of IOP fluctuation was 2.08 mmHg. Patients with SD of IOP ≥ 2.08 mmHg were classified into the high IOP fluctuation group, and those with SD of IOP < 2.08 mmHg into the low IOP fluctuation group.

Demographics are displayed in Table 1. Patients with ERM (62.7 ± 7.6 years) were older than those without ERM (58.0 ± 10.1 years, *p* = 0.018). Mean IOP was similar between groups (*p* = 0.473). Lens status showed that the proportion of pseudophakia was higher in patients with ERM than in those without it (*p* = 0.013). The SD of IOP was higher in the ERM group (2.2 ± 0.7 mmHg) than in the non-ERM group (1.9 ± 0.7 mmHg, *p* = 0.049). The proportion of high IOP fluctuation was greater in patients with ERM (61.0%) than in those without ERM (34.9%, *p* = 0.028). Macular thickness was greater in the ERM group than in the non-ERM group (*p* < 0.001). Patients with ERM had thinner superior RNFL thickness than those without ERM (*p* = 0.011). Patients with ERM had a higher probability of having microcystic macular changes than those without ERM (*p* = 0.005). There was no significant difference in SD of IOP according to the presence of microcystic macular changes (*p* = 0.082), even though patients with microcystic macular changes showed higher SD of IOP (2.5 ± 0.8 mmHg) than those without microcystic macular changes (2.0 ± 0.7 mmHg).

The high fluctuation group showed a greater proportion of ERM and greater macular thickness than the low IOP fluctuation group (*p* = 0.028 and *p* = 0.005, Table 2).

In the subgroup analysis on subjects with mean IOP < 15 mmHg, the proportion of patients with high IOP fluctuation was greater in eyes with ERM than those without ERM (29.0%) (*p* = 0.021, Table 3). Analysis of lens status found that eyes with ERM showed a greater proportion of pseudophakia than those without ERM (*p* = 0.040).

In multiple logistic regression analysis, only high IOP fluctuation was related to the presence of ERM (*p* = 0.047, Table 4). In patients with mean IOP < 15 mmHg, high IOP fluctuation was associated with ERM (*p* = 0.031).

In subgroup analysis in patients with ERM, the late ERM group (stage 3 or 4) showed greater macular thickness than the early ERM group (stage 1 or 2) (*p* < 0.001, Table 5). The MD of VF 24-2 was lower in patients with later-stage ERM (MD = −13.7 ± 9.6 dB) than in those with early stage ERM (−7.7 ± 6.8 dB, *p* = 0.025).

In patients with available HRV data, the high IOP fluctuation group showed a higher LF value of HRV, which is considered to be a quantitative marker for sympathetic modulations, than the low IOP fluctuation group (*p* = 0.027, Table 6).

The SD of IOP was positively correlated with the LF component of HRV (r = 0.503, *p* = 0.033, Table 7).

Intereye comparison in eyes with ERM and the fellow eyes without ERM showed that eyes with ERM (2.2 ± 0.9 mmHg) had a higher fluctuation of IOP than those without ERM (2.0 ± 0.8 mmHg, *p* = 0.029, Table 8). The trough of IOP was lower in eyes with ERM than in the fellow eyes (*p* = 0.026). Macular thickness was greater in eyes with ERM than in the fellow eyes (*p* < 0.001).

**Figure 1:**
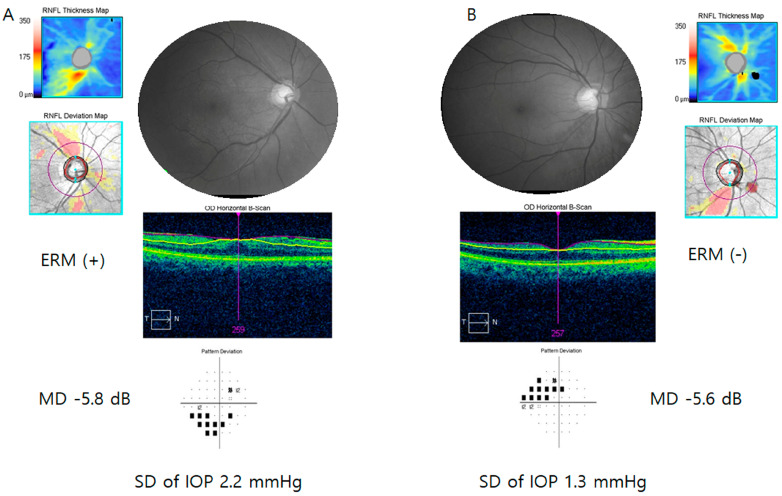
(**A**) A female ages 50–60 years old had glaucoma and epiretinal membrane (ERM) in her right eye. Mean deviation (MD) of visual field test was −5.8 dB. (**B**) A female ages 50–60 years had glaucoma (MD = −5.6 dB) without ERM in her right eye. Standard deviation (SD) of intraocular pressure was greater in the patient with ERM ((**A**): 2.2 mmHg) than subject without ERM ((**B**): 1.3 mmHg).

Representative cases are shown in Figure 1. A female aged 50–60 years had glaucoma (MD = −5.8 dB) and ERM in her right eye. A female ages 50–60 years had glaucoma (MD = −5.6 dB) and showed no signs of ERM in her right eye. IOP fluctuation was greater in the patient with ERM (A, SD of IOP: 2.2 mmHg) than in the subject without ERM (B, SD of IOP = 1.3 mmHg).

**Figure 2:**
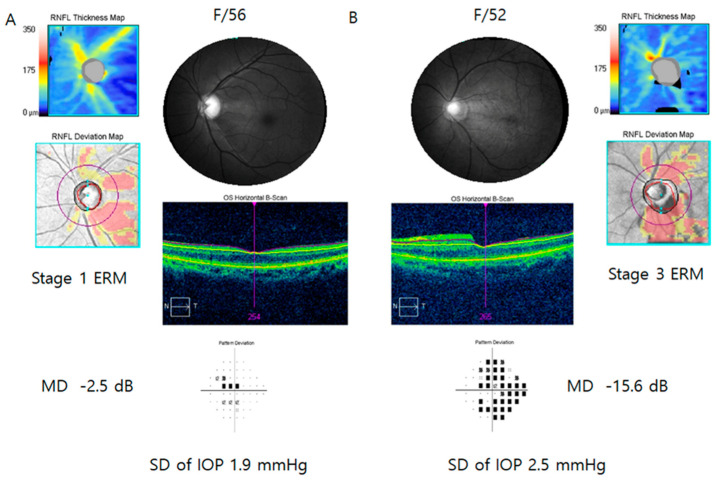
Representative cases showing the difference in glaucomatous visual field damage according to epiretinal membrane (ERM) stage (**A**) Fifty-six-year-old patient showed glaucoma and stage 1 ERM. Standard deviation (SD) of intraocular pressure (IOP) was 1.9 mmHg. (**B**) Fifty-two-year-old female had stage 3 ERM with glaucoma. SD of IOP was 2.5 mmHg. The patient with advanced stage ERM ((**B**), mean deviation of visual field test = −15.6 dB) had more severe glaucomatous visual field damage than the patient with mild stage ERM ((**A**), mean deviation of visual field test = −2.5 dB).

Figure 2 represents two patients with glaucoma and ERM. The patient with stage 3 ERM (B, mean deviation of visual field test = −15.6 dB) had more severe glaucomatous visual field damage than the patient with stage 1 ERM (A, mean deviation of visual field test = −2.5 dB).

## 4. Discussion

We demonstrated that among patients with glaucoma or glaucoma suspect, those with idiopathic ERM were older and had higher IOP fluctuation and a higher proportion of history of cataract operation than those without ERM. In multiple logistic regression analysis, only the high IOP fluctuation group was significantly associated with presence of ERM. Intereye comparison in patients with unilateral ERM showed that eyes with ERM showed higher IOP fluctuation compared to fellow eyes without ERM.

Among patients with glaucoma or glaucoma suspect, we found that high IOP fluctuation was associated with high probability of having idiopathic ERM. Intereye analysis also demonstrated that eyes with ERM (2.2 ± 0.9 mmHg) had higher fluctuation of IOP than those without ERM (2.0 ± 0.8 mmHg, *p* = 0.029). Previously, our group reported that greater IOP fluctuation was related to the development of early stage (grade 1 or 2) of ERMs [31]. Several estimates might explain the correlation between greater IOP fluctuation and ERM. Previous history of cataract surgery is one of risk factors for development of idiopathic ERM [5,32]. In this study, glaucoma patients with ERM showed a higher proportion of eyes with a history of cataract surgery than those without ERM (*p* = 0.013), even though the correlation did not remain in multiple logistic regression analysis (*p* = 0.104). Anteroposterior movement of the vitreous leading to dynamic traction at the posterior border of the vitreous has been suggested to be frequently associated with the pathogenesis of PVD and subsequent occurrence of ERM [33]. During cataract surgery, IOP could abruptly increase or decrease when the tip of the phacoemulsification device is inserted into or removed from the anterior chamber or when the lens is removed. Large IOP fluctuation might induce forward and backward movement of the vitreous, leading to the development of PVD and subsequent ERM. First, we assumed that anteroposterior movement of the vitreous and subsequent PVD induced by greater IOP fluctuation might be one of the reasons why large IOP fluctuation could be related to the presence of ERM. Second, large IOP fluctuations could lead to gliosis, reactive proliferation of glial cells, being one of the components that participate in formation of ERM [21,22]. There is a possibility that IOP fluctuations have an association with gliosis in the retina because activation of astrocyte reactivity was observed in the optic nerve head of rats undergoing intermittent IOP elevations during six weeks [20]. In the subgroup analysis on subjects with mean IOP < 15 mmHg, the proportion of patients with high IOP fluctuation was greater in eyes with ERM than those without ERM (*p* = 0.021). Given those findings, greater IOP fluctuation could be more significant than the higher IOP value itself regarding association between IOP and ERM.

In terms of ERM severity, eyes with stage 3 or 4 ERM had more advanced VF damage than those with stage 1 or 2 ERM. That result corresponds to Sakimoto et al.’s study which found that glaucoma patients with unilateral ERM showed more severe glaucomatous VF damage and more glaucomatous optic disc change in eyes with ERM than in fellow eyes [6]. Lee et al. also reported that the presence of ERM was significantly related to worse VF loss in eyes with pseudoexfoliative glaucoma [8]. Sakimoto and associates speculated that the mechanical stress caused by inner retinal traction induced by ERM might induce damage of the RNFL or the inner plexiform layer, especially in eyes with vulnerable and dysfunctional retinal ganglion cells in patients with glaucoma [6]. In patients with advanced-stage glaucoma, the measurements of RNFL thickness using OCT shows a persistent residual layer, beyond which no further thinning is observed [34,35]. Proliferation of glial cells in the human retina and optic nerve head after glaucomatous retinal ganglion cell damage is one plausible explanation for the floor effect of OCT, although this phenomenon might be explained by the presence of blood vessels [34,36,37]. Therefore, gliosis after substantial loss of retinal ganglion cells in glaucoma patients might stimulate formation of ERM, although more studies are needed to prove it.

Old age is one of the well-known factors associated with the presence of ERM [3,4,32]. The correlation between aging and ERM could be related to the findings that PVD develops increasingly with age [32,38]. When PVD occurs, vitreomacular traction triggers defects in the internal limiting membrane, and cellular components such as glial cells migrate to defects in the retinal surface, leading to ERM formation [32,38]. This study did not find any correlation between old age and ERM in multiple logistic regression analysis, although a correlation was observed in univariate analysis. We speculated that IOP fluctuation played a greater role in patients with both glaucoma and ERM.

IOP is controlled by the velocity of both aqueous humor production and outflow. Aqueous humor production in the ciliary processes and outflow through the trabecular meshwork and episcleral venous vasculature are influenced by the autonomic nervous system in some degree [23]. Dysfunction of autonomic control was frequently found in patients with NTG [39]. Patients with primary generalized autonomic failure had substantial posture-related changes in IOP, which were greater than the changes seen in normal control subjects [40]. In this study, subgroup analysis found that the high IOP fluctuation group had a higher LF value of HRV than the low IOP fluctuation group. There was also a positive correlation between the LF of HRV and the SD of IOP. The LF component is indicated as a quantitative marker for sympathetic modulations, although there is a controversy on its origin [41]. Therefore, abnormal autonomic control might affect greater IOP fluctuation in glaucoma patients.

One of the limitations in this study is that the relationship between IOP fluctuation and idiopathic ERM was analyzed only in patients with glaucoma or glaucoma suspect. Therefore, findings from this study should not be generalized to patients without glaucoma or glaucoma suspect. Further studies are required to investigate the relationship between IOP variability and idiopathic ERM in subjects without glaucoma. The control group, comprised of patients without ERM and with glaucoma or glaucoma suspect, was enrolled within a relatively short time frame than those with ERM. While this limited recruitment period could potentially affect the generalizability of our findings, there was no statistically difference in the follow-up period between the groups. A substantial portion of patients with glaucoma or glaucoma suspect (85.7%) were using glaucoma eyedrops. However, the proportion of patients using glaucoma eyedrops was not significantly different between patients with and without ERM, even though the effects of glaucoma eyedrops on the results were not totally excluded.

## 5. Conclusions

In conclusion, glaucoma patients with ERM had a higher probability of having greater IOP fluctuation. Determination of long-term IOP fluctuation can take a relatively long time compared to detection of ERM when physicians see a patient. ERM could be a biomarker for long-term IOP fluctuation in patients with glaucoma. Patients with a more severe stage of ERM had a more advanced stage of glaucoma. Patients with advanced stage of both ERM and glaucoma could have definitely decreased visual function.

## Figures and Tables

**Table 1:** Comparison of patients with glaucoma or glaucoma suspect according to the presence of epiretinal membrane.

	ERM (−) (*n* = 43)	ERM (+) (*n* = 41)	*p* Value
Age (years)	58.0 ± 10.1	62.7 ± 7.6	**0.018**
Male/Female	17/26	15/26	0.825
Diabetes (%)	4 (9.3%)	5 (12.2%)	0.735
Systemic hypertension (%)	16 (37.2%)	18 (52.9%)	0.657
Migraine (%)	4 (9.3%)	6 (14.6%)	0.515
Cold hand (%)	5 (11.6%)	4 (9.8%)	1.000
Thyroid disease	1 (2.3%)	0 (0%)	
Systolic blood pressure (mmHg)	127.9 ± 15.4	128.7 ± 13.0	0.856
Diastolic blood pressure (mmHg)	80.8 ± 9.1	80.1 ± 9.5	0.788
Lens status (phakic/pseudophakic)	38/5 (88.4%/11.6%)	27/14 (65.9%/34.1%)	**0.013**
Central corneal thickness (µm)	524.1 ± 33.1	525.9 ± 34.6	0.829
Spherical equivalent (diopter)	−1.5 ± 3.1	−1.5 ± 2.8	0.954
Axial length (mm)	24.2 ± 1.4	23.8 ± 1.2	0.300
Mean IOP (mmHg)	13.9 ± 2.2	13.6 ± 2.1	0.473
SD of IOP (mmHg)	1.9 ± 0.7	2.2 ± 0.7	**0.049**
Peak IOP (mmHg)	17.5 ± 3.3	17.9 ± 4.3	0.585
Trough of IOP (mmHg)	10.7 ± 2.2	10.5 ± 2.1	0.557
Range of IOP (Peak-trough) (mmHg)	6.7 ± 3.1	7.5 ± 4.0	0.345
Presence of disc hemorrhage (%)	7 (16.3%)	2 (4.9%)	0.157
Number of high IOP fluctuation group (%)	15 (34.9%)	25 (61.0%)	**0.028**
Macular thickness (µm)	248.0 ± 27.8	344.4 ± 74.2	**<0.001**
Presence of microcystic marcular changes	0 (0%)	7 (17.1%)	**0.005**
Average RNFL thickness (µm)	69.9 ± 12.8	67.8 ± 10.7	0.434
Number of glaucoma eyedrops	1.4 ± 0.9	1.6 ± 0.9	0.270
MD of VF 24-2 (dB)	−8.3 ± 7.4	−9.8 ± 8.3	0.396
PSD of VF 24-2 (dB)	7.8 ± 4.6	7.7 ± 4.3	0.972
Follow-up period (months)	100.2 ± 42.4	92.6 ± 39.7	0.404

ERM, epiretinal membrane; IOP, intraocular pressure; MD, mean deviation; PSD, pattern standard deviation; RNFL, retinal nerve fiber layer; VF, visual field. Statistically significant differences between two groups (*p* < 0.05) by student’s *t*-test for continuous variables or chi-squared test for categorical data are indicated in bold.

**Table 2:** Characteristics of patients with glaucoma or glaucoma suspect in each intraocular pressure (IOP) fluctuation group.

	Low IOP Fluctuation Group (SD of IOP < 2.08 mmHg) (*n* = 44)	High IOP Fluctuation Group (SD of IOP ≥ 2.08 mmHg) (*n* = 40)	*p* Value
Age (years)	58.8 ± 8.5	61.9 ± 9.7	0.124
Male/Female	16/28	16/24	0.823
Diabetes (%)	5 (11.4%)	4 (10.0%)	1.000
Systemic hypertension (%)	19 (43.2%)	15 (37.5%)	0.660
Migraine (%)	7 (15.9%)	3 (7.5%)	0.319
Cold hand (%)	4 (9.1%)	5 (12.5%)	0.730
Thyroid disease	1 (2.3%)	0	1.000
Systolic blood pressure (mmHg)	127.0 ± 15.1	130.2 ± 13.2	0.461
Diastolic blood pressure (mmHg)	79.9 ± 8.8	81.4 ± 10.0	0.572
Lens status (phakic/pseudophakic)	36/8 (81.8%/18.2%)	29/11 (72.5%/27.5%)	0.434
Central corneal thickness (µm)	520.8 ± 33.7	530.5 ± 33.3	0.223
Spherical equivalent (diopter)	−1.3 ± 3.0	−1.6 ± 2.9	0.618
Axial length (mm)	24.0 ± 1.2	24.1 ± 1.4	0.873
Mean IOP (mmHg)	13.4 ± 2.2	14.2 ± 2.0	0.076
SD of IOP (mmHg)	1.5 ± 0.4	2.6 ± 0.4	**<0.001**
Peak IOP (mmHg)	16.2 ± 3.1	19.3 ± 3.9	**<0.001**
Trough of IOP (mmHg)	10.8 ± 2.2	10.4 ± 2.2	0.304
Range of IOP (Peak-trough) (mmHg)	5.4 ± 2.5	9.0 ± 3.6	**<0.001**
Presence of disc hemorrhage (%)	3 (6.8%)	6 (15.0%)	0.298
Presence of ERM (%)	36.4%	62.5%	**0.028**
Macular thickness (µm)	272.6 ± 62.8	317.9 ± 77.1	**0.005**
Average RNFL thickness (µm)	71.3 ± 10.7	66.2 ± 12.5	0.050
Number of glaucoma eyedrops	1.3 ± 0.9	1.7 ± 0.8	0.084
MD of VF 24-2 (dB)	−7.4 ± 7.2	−10.8 ± 8.2	0.050
PSD of VF 24-2 (dB)	7.4 ± 4.5	8.1 ± 4.4	0.475

ERM, epiretinal membrane; IOP, intraocular pressure; MD, mean deviation; PSD, pattern standard deviation; RNFL, retinal nerve fiber layer; VF, visual field. Statistically significant differences between two groups (*p* < 0.05) by student’s *t*-test for continuous variables or chi-squared test for categorical data are indicated in bold.

**Table 3:** Subgroup analysis in lower mean and higher mean intraocular pressure (IOP) group divided based on mean IOP of 15 mmHg.

	Mean IOP < 15 mmHg (*n* = 61)			Mean IOP ≥ 15 mmHg (*n* = 23)		
	ERM (−) (*n* = 31)	ERM (+) (*n* = 30)	*p* Value	ERM (−) (*n* = 12)	ERM (+) (*n* = 11)	*p* Value
Age (years)	58.2 ± 9.5	62.9 ± 7.7	**0.037**	57.4.11.9	62.0 ± 7.6	0.287
Male/Female	13/18	11/19	0.795	4/8	4/7	1.000
Diabetes (%)	4 (12.9%)	5 (16.7%)	0.731	0	0	N/A
Systemic hypertension(%)	13 (41.9%)	15 (50.0%)	0.611	3 (25.0%)	3 (27.3%)	1.000
Migraine(%)	3 (9.7%)	5 (16.7%)	0.473	1 (8.3%)	1 (9.1%)	1.000
Cold hand(%)	5 (16.1%)	4 (13.3%)	1.000	0	0	N/A
Thyroid disease	1 (3.2%)	0	1.000	0	0	N/A
Systolic BP(mmHg)	128.2 ± 18.0	129.1 ± 13.7	0.869	127.3 ± 8.5	127.0 ± 11.0	0.953
Diastolic BP (mmHg)	81.3 ± 10.1	80.7 ± 9.5	0.839	80.0 ± 7.2	77.5 ± 10.5	0.685
Lens status (phakic/pseudophakic)	27/4 (87.1%/12.9%)	19/11 (63.9%/36.7%)	**0.040**	11/1 (91.7%/8.3%)	8/3 (72.7%/27.3%)	0.261
Central corneal thickness (µm)	517.5 ± 30.9	521.8 ± 31.7	0.608	543.6 ± 32.9	537.6 ± 41.9	0.352
Spherical equivalent (diopter)	−1.6 ± 3.0	−1.7 ± 2.8	0.886	−1.2 ± 3.4	−0.8 ± 2.7	0.730
Axial length (mm)	24.2 ± 1.2	24.0 ± 1.0	0.534	24.2 ± 1.8	23.4 ± 1.5	0.385
Mean IOP (mmHg)	12.9 ± 1.7	12.7 ± 1.7	0.532	16.4 ± 9.3	16.1 ± 0.7	0.285
SD of IOP (mmHg)	1.9 ± 0.7	2.1 ± 0.7	0.165	2.0 ± 0.7	2.3 ± 0.6	0.145
Presence of disc hemorrhage (%)	7 (22.6%)	2 (6.7%)	0.147	0	0	N/A
Number of high IOP fluctuation group (%)	9 (29.0%)	18 (60.0%)	**0.021**	6 (50%)	7 (63.6%)	0.680
Average RNFL thickness (µm)	71.3 ± 13.3	67.0 ± 11.1	0.173	66.1 ± 11.0	70.1 ± 9.9	0.373
Number of glaucoma eyedrops	1.4 ± 1.0	1.6 ± 0.9	0.302	1.4 ± 0.8	1.5 ± 0.8	0.706
MD of VF 24-2 (dB)	−9.0 ± 8.3	−10.0 ± 8.9	0.666	−6.5 ± 3.9	−9.2 ± 3.8	0.244
PSD of VF 24-2 (dB)	7.5 ± 4.6	7.8 ± 4.4	0.806	8.4 ± 4.7	7.5 ± 4.3	0.648

BP, blood pressure; ERM, epiretinal membrane; IOP, intraocular pressure; MD, mean deviation; PSD, pattern standard deviation; RNFL, retinal nerve fiber layer; VF, visual field Statistically significant differences between two groups (*p* < 0.05) by student’s *t*-test for continuous variables or chi-squared test for categorical data are indicated in bold.

**Table 4:** Multiple logistic regression analysis of factors associated with the presence of epiretinal membrane in patients with glaucoma suspect or glaucoma.

Parameter	Total Patients		Mean IOP < 15 mmHg		Mean IOP ≥ 15 mmHg	
	β Coefficient (95% CI)	*p* Value	β Coefficient (95% CI)	*p* Value	β Coefficient (95% CI)	*p* Value
**Age**	1.039 (0.981–1.101)	0.194	1.036 (0.962–1.116)	0.346	1.051 (0.950–1.163)	0.316
**Lens status**	2.781 (0.809–9.559)	0.104	2.641 (0.577–12.076)	0.211	4.085 (0.339–49.175)	0.268
**High IOP fluctuation group**	2.573 (1.015–6.525)	**0.047**	3.379 (1.116–10.363)	**0.031**	1.459 (0.260–8.198)	0.827

IOP, intraocular pressure. The variables that was statistically significant in the multiple logistic regression analysis was indicated in bold.

**Table 5:** Subgroup analysis according to severity of epiretinal membrane in patients with epiretinal membrane and glaucoma suspect or glaucoma.

	Early ERM Stage (1 or 2) (*n* = 27)	Late ERM Stage (3 or 4) (*n* = 14)	*p* Value
Age (years)	61.9 ± 7.0	64.2 ± 8.8	0.352
Male/Female	12/15	3/11	0.133
Diabetes (%)	2 (7.4%)	3 (21.4%)	0.317
Systemic hypertension(%)	10 (37.0%)	8 (57.1%)	0.322
Migraine(%)	6 (22.2%)	0 (0%)	0.079
Cold hand(%)	2 (7.4%)	2 (14.3%)	0.596
Thyroid disease	0 (0%)	0 (0%)	N/A
Systolic blood pressure	130.5 ± 14.3	124.5 ± 8.7	0.357
Diastolic blood pressure	82.1 ± 10.3	75.2 ± 5.2	0.137
Lens status (phakic/pseudophakic)	19/8 (70.4%/29.6%)	8/6 (57.1%/42.9%)	0.494
Central corneal thickness (µm)	519.4 ± 29.8	542.1 ± 41.9	0.079
Spherical equivalent (diopter)	−1.7 ± 3.1	−1.1 ± 1.9	0.531
Axial length (mm)	24.0 ± 1.3	23.6 ± 0.9	0.373
Mean IOP (mmHg)	13.8 ± 2.0	13.1 ± 2.4	0.356
SD of IOP (mmHg)	2.2 ± 0.7	2.1 ± 0.5	0.652
Peak IOP (mmHg)	18.3 ± 4.9	17.3 ± 3.2	0.503
Trough of IOP (mmHg)	10.4 ± 2.0	10.5 ± 2.4	0.938
Range of IOP (Peak-trough) (mmHg)	7.8 ± 4.7	6.8 ± 2.0	0.443
Presence of disc hemorrhage (%)	0 (0%)	2 (14.3%)	0.111
Number of high IOP fluctuation group (%)	15 (55.6%)	10 (71.4%)	0.501
Macular thickness (µm)	308.9 ± 51.6	415.2 ± 61.3	**<0.001**
Average RNFL thickness (µm)	68.9 ± 11.8	65.9 ± 8.4	0.404
Number of glaucoma eyedrops	1.6 ± 0.9	1.6 ± 0.9	0.841
MD of VF 24-2 (dB)	−7.7 ± 6.8	−13.7 ± 9.6	**0.025**
PSD of VF 24-2 (dB)	7.6 ± 4.7	8.1 ± 3.6	0.725

ERM, epiretinal membrane; MD, mean deviation; PSD, pattern standard deviation; SAP, standard automated perimetry. Statistically significant differences between two groups (*p* < 0.05) by student’s *t*-test for continuous variables or chi-squared test for categorical data are indicated in bold.

**Table 6:** Subgroup analysis in low and high IOP fluctuation group among patients with available heart rate variability (HRV) data.

HRV Data	Low IOP Fluctuation Group (SD of IOP < 2.08 mmHg) (*n* = 10)	High IOP Fluctuation Group (SD of IOP ≥ 2.08 mmHg) (*n* = 8)	*p* Value
SDNN	28.8 ± 17.3	38.8 ± 12.8	0.083
RMSSD	21.7 ± 18.1	30.4 ± 17.6	0.146
LF	256.8 ± 472.3	751.3 ± 635.4	**0.027**
HF	135.8 ± 248.8	271.2 ± 267.8	0.068
LF/HF	1.5 ± 1.2	6.3 ± 8.0	0.146

Mann–Whitney U Test. HF, high frequency; HRV, heart rate variability; LF, low frequency; RMSSD, root mean square successive difference; SDNN, standard deviation of the NN-interval.

**Table 7:** The relationship between standard deviation of IOP and heart rate variability (HRV) among patients with available HRV data.

HRV Data	r	*p* Value
SDNN	0.338	0.171
RMSSD	0.222	0.377
LF	0.503	**0.033**
HF	0.292	0.240
LF/HF	0.439	0.068

HF, high frequency; HRV, heart rate variability; LF, low frequency; RMSSD, root mean square successive difference; SDNN, standard deviation of the NN-interval; r, Spearman’s rho.

**Table 8:** Intereye comparison in eyes with an ERM and the fellow eyes without an epiretinal membrane (ERM) in patients with glaucoma or glaucoma suspect.

	ERM (−) (*n* = 28)	ERM (+) (*n* = 28)	*p* Value
Lens status (phakic/pseudophakic)	23/5 (82.1%/17.9%)	21/7 (75.0%/25.0%)	0.500†
Mean IOP (mmHg)	13.8 ± 2.4	13.4 ± 2.3	0.077 *
SD of IOP (mmHg)	2.0 ± 0.8	2.2 ± 0.9	0.029 *
Peak IOP (mmHg)	17.4 ± 3.9	17.6 ± 5.0	0.863 *
Trough of IOP (mmHg)	10.5 ± 2.3	9.8 ± 1.9	0.026 *
Range of IOP (Peak-trough) (mmHg)	7.0 ± 3.4	7.7 ± 4.8	0.200 *
Central corneal thickness (µm)	519.2 ± 32.5	514.8 ± 31.4	0.035 *
Spherical equivalent (diopter)	−1.3 ± 3.0	−1.1 ± 3.0	0.838 *
Axial length (mm)	23.9 ± 1.3	23.8 ± 1.4	0.643 *
Number of high IOP fluctuation group (%) †	11 (39.3%)	14 (50.0%)	0.508 †
Macular thickness (µm)	247.1 ± 29.4	333.2 ± 72.6	<0.001 *
Average RNFL thickness (µm)	69.4 ± 13.3	70.8 ± 12.1	0.859 *
Number of glaucoma eyedrops	1.4 ± 1.0	1.6 ± 0.9	0.056 *
MD of VF 24-2 (dB)	−8.5 ± 8.8	−8.3 ± 7.4	0.872 *
PSD of VF 24-2 (dB)	6.5 ± 4.2	7.1 ± 4.8	0.448 *

ERM, epiretinal membrane; MD, mean deviation; PSD, pattern standard deviation; SAP, standard automated perimetry. The variables that was statistically significant in * Wilcoxon signed rank test and †: McNemar are indicated in bold.

## Data Availability

Data are available upon reasonable request to the corresponding author.
